# A Case of Facial Erysipelas in a Lying-in Patient without Puerperal Fever

**Published:** 1882-01

**Authors:** Norman Bridge

**Affiliations:** 81 Throop St.; Chicago


CHICAGO MEDICAL

JOURNAL & EXAMINER.

VOL. XLIV.—JANUARY, 1882.—No. 1.

### Original Communications.

ARTICLE I.

### A CASE OF FACIAL ERYSIPELAS IN A LYING-IN PATIENT WITHOUT PUERPERAL FEVER. By NORMAN BRIDGE, M.D., Chicago.

The report of a case of "erysipelas in child-bed without puerperal peritonitis," at the recent meeting of the American Gynæcological Society, by Dr. Campbell, of Georgia, has led me to recall a similar case in my own practice, and to examine the notes taken at that time.

The patient was a woman of about forty years of age, who had been something of an invalid for several years; who was exceedingly nervous, irritable, dyspeptic and despondent. For weeks previous to confinement she suffered with various neuralgic pains, vomited most of the little food she ate, and thoroughly believed she would die in her confinement. Her last previous confinement, eight years before, had been a severe one; had resulted in an extensive laceration of the perineum, for the relief of which Prof. Peaslee had, a year before, made a successful operation.

Her confinement occurred in the night, was very rapid, and delivery was accomplished about half an hour before my arrival. I found the child dead, but it was said to have been alive at birth, and to have cried. It had not been removed from the mother. I delivered the placenta, and soon afterward left the patient in a comfortable condition.

At my call the next day, at eleven o'clock, the patient complained of a burning sensation on her nose; a small spot of erysipelas was noticeable on that part of the nose where facial erysipelas so often begins, and her pulse was found to be 84, and her temperature 102° F. She complained of headache.

The erysipelatous surface was painted with flexible collodion; hot, carbolized, vaginal injections were ordered, and sulphate of cinchonidia to be taken—two grains every two hours. At five P. M. the only changes noted were a rise of half a degree in the temperature, and a rapid spreading of the erysipelas. At one P. M. the next day the disease had covered both cheeks and under eyelids, upper lip, and the entire surface of the nose. The pharynx was inflamed, and the patient complained of soreness in swallowing. No change in temperature; pulse 80.

The next day (the third after confinement) the forehead, part of the scalp and ears had become involved in the inflammation. The tincture of iron was now ordered in small doses, but soon had to be abandoned, on account of the gastric irritability. Pulse 84; temperature 102¼.

On the following day at five P. M. the pulse was 90, and full, the temperature was 102 3-5° F., and there was now discovered, for the first time, slight tenderness over the region of the uterus. No tympanitis was present. The lochia, which till now had been free from bad odor, were slightly fetid. This symptom had disappeared by the next day, and the abdominal tenderness was nearly gone. The temperature had fallen to 102 1-5, and the pulse to 80. The erysipelas had ceased to spread, and was fading away upon the central portions of the face. Recovery now was rapid. There were no other unfavorable symptoms; the erysipelas recovered in the usual time and manner, and there was free exfoliation of the cuticle.

In ten days after the confinement the patient was ready to begin to assume the sitting posture, as though no complication had occurred. The cinchonidia sulphate was taken quite regularly during the sickness, the vaginal douches were not omitted, and the face was daily painted with the flexible collodion.

There was no delirium at any time.

81 Throop St., Dec., 1881.

